# The effect of compression therapies and therapeutic modalities on lymphedema secondary to cancer: a rapid review and evidence map

**DOI:** 10.1007/s12032-024-02447-w

**Published:** 2024-10-17

**Authors:** M. L. McNeely, S. M. Shallwani, M. M. Al Onazi, F. Lurie

**Affiliations:** 1 Department of Physical Therapy, University of Alberta, Edmonton, AB Canada; 2 Cancer Care Alberta, Alberta Health Services, Edmonton, AB Canada; 3 Department of Vascular Surgery, Promedica Jobst Vascular Institute, Toledo, OH USA

**Keywords:** Cancer, Lymphedema, Compression therapies, Compression pumps, Low-level laser therapy, Extracorporeal shockwave therapy, Mapping review

## Abstract

The identification of effective therapeutic modalities to manage lymphedema secondary to cancer is a high priority among patients and clinicians. Complex decongestive therapy (CDT) remains a fundamental intervention for individuals with lymphedema; however, interventions involving modalities such as low level laser therapy, specially designed compression systems, and compression pumps may be helpful to improve outcomes and reduce costs of care. We conducted a rapid review of the literature examining compression therapies and therapeutic modalities in the treatment of lymphedema secondary to cancer. A search of the electronic databases from June 2018 to October 2023 was performed including MEDLINE, EMBASE, and CINAHL. The electronic search yielded 438 potentially relevant citations with 40 randomized controlled trials included in the review, and 30 in the mapping process. Ninety-three percent (n = 37) of the trials included participants with a diagnosis of breast cancer. Across all categories and domains, all but two trials were rated as having ‘some concerns’ or a ‘high risk of bias’. Intervention effects ranged from clinically insignificant to large effects on lymphedema volume. Evidence mapping suggests potential for benefit from (1) compression garments for the prevention of lymphedema, (2) interventions added to CDT in the intensive reduction phase, and (3) nighttime compression and compression pump treatments in the maintenance phase. A multi-centre collaborative research approach is needed to support the conduct of high-quality large-scale trials to inform the optimal type, timing, and combination of compression therapies and therapeutic modalities in the treatment of lymphedema secondary to cancer.

**Supplementary Information:**

The online version contains supplementary material available at 10.1007/s12032-024-02447-w.

## Introduction

Lymphedema is a chronic and progressive disease of the lymphatic system characterized by progressive swelling of the affected regions of the body, chronic inflammation, and increased deposition of fibroadipose tissue [1]. In North America, cancer-related treatments such as surgery and radiation therapy are leading causes of secondary lymphedema [1]. Lymphedema affects approximately one in five individuals with cancer, causing pain, functional limitation, distortion of limb shape, and repeated limb infections [2]. Currently, there is no cure for lymphedema. The financial burden of managing lymphedema to patients, payers, and society is significant [3]. Over time, chronic lymphedema negatively influences the individual’s physiological, psychological, and social functioning [4–6].

The identification of effective therapeutic modalities to manage this condition remains a high priority among both patients and clinicians [7]. Treatment options for lymphedema aim to reduce excess fluid volume in the limb or body region and improve associated symptoms [8]. Currently, complex decongestive therapy (CDT) is the gold standard for the conservative treatment of lymphedema [2] and involves two phases, an intensive reduction phase followed by a self-management maintenance phase that aims to stabilize the condition [2]. The intensive phase of CDT consists of manual lymph drainage (i.e., a specialized massage technique for the treatment of lymphedema), multi-component compression bandaging, skin care, and decongestive exercises [2]. The maintenance phase involves use of a daytime compression garment, skin care, and decongestive exercises, with additional reduction interventions administered as needed.

Beyond standard CDT components, other treatment modalities for lymphedema can be prescribed in either reduction or maintenance phases of treatment. Compression pumps are commonly used in the treatment of lymphedema and serve as an option that can be self-administered at home [9, 10]. Evidence suggests that the intermittent compression applied by a compression pump leads to the formation of tissue channels that can aid in the removal of fluid [9]. Low level laser therapy targets cellular mechanisms to help reduce inflammation and enhance immune system functioning and is proposed as a method to facilitate the drainage of excess protein-rich fluid [11]. Extracorporeal shock wave therapy emits acoustic waves (shock waves) that can trigger interstitial and extracellular responses. Extracorporeal shock wave therapy is proposed to help relieve pain and inflammation, and prevent soft tissue fibrosis [12]. Alternative forms of compression therapies, such as non-elastic Velcro wraps, day and nighttime compression systems, and elastic taping (e.g., Kinesio®, KT) are often preferred due to their comfort and ease in application [13].

Several systematic reviews (SRs) have been published; however, it is unclear if and when any of these treatment modalities has a more relevant effect [4]. A recent overview of interventions for breast cancer related lymphedema (BCRL) involved 18 SRs with meta-analyses involving 51 randomized controlled trials (RCTs) [8]. The findings suggest no evidence of superiority of any one intervention for the outcome of lymphedema volume nor any evidence refuting any specific intervention [8]. The meta-analysis showed only small effects from interventions when the data were pooled, comparing all experimental interventions to any control/comparison intervention. The authors identified several challenges including the high risk of bias of the included trials, and the heterogeneity among trials in inclusion criteria for BCRL, treatment regimens, and study populations. While this overview provides an estimate suggesting a small effect from interventions for lymphedema, the high heterogeneity among studies in experimental as well as in control and comparison interventions limits confidence in the findings.

A systematic evidence map is an evidence synthesis method that is used to visually display study characteristics and findings [14]. An evidence map can show what has been studied, what is known, and where there are gaps in the research. Evidence mapping is used to guide clinical investigators and can help to set the agenda for future research [14]. The aim of the present review was to map the research evidence from RCTs examining the benefit of compression therapies and therapeutic modalities for lymphedema in individuals with lymphedema secondary to cancer. Our goal was to identify knowledge gaps in the field of cancer-related lymphedema to inform future research.

## Methods

This research area was identified as a priority topic for the 2023 Lymphedema Summit: Forward Momentum; Future Steps in Lymphedema Management. To produce information within the time and resource constraints of the Lymphedema Summit, we conducted a systematic mapping review using a rapid search method of the literature, limited to the last 5 years [15]. We aimed to rigorously review SRs (with or without meta-analyses), as well as recent RCTs examining compression therapies and therapeutic modalities in the treatment of lymphedema secondary to cancer.

### Mapping review question

Do compression therapies and therapeutic modalities improve lymphedema incidence and volume in individuals with lymphedema secondary to cancer?

### The objectives of the mapping review were to

- Appraise the evidence from eligible RCTs that were recently published or included in a recent SR, which focused on compression therapies and therapeutic modalities in the treatment of lymphedema secondary to cancer.
- Produce a visual evidence map to synthesize findings for the outcome of lymphedema volume/incidence and inform the current state of the research evidence.

### Data sources and search strategy

A search of the electronic databases from June 2018 to October 2023 was performed including MEDLINE, EMBASE, and CINAHL. The research team developed the search strategy with support from an expert reference librarian. We used search terms related to cancer (e.g. neoplasms, lymph node excision), lymphedema (e.g. lymphedema, lymphoedema, edema), compression treatments (e.g., stockings, compression, night-time compression systems, bandaging, elastic taping), devices (e.g., low level laser therapy, extracorporeal shock wave therapy), compression pumps (e.g., Lymphapress, Flexitouch) and publication type (e.g., SR, meta-analysis, RCT). Only published reviews and trials, restricted to English language, were eligible for inclusion. The detailed search strategy is provided in the online supplementary material: Appendix A.

### Eligibility criteria

We included any recent SR or RCT that:

1. Enrolled adults (where at least 90% of the study population were aged 18 years or older) with or at risk of lymphedema secondary to cancer.
2. Included an intervention(s) involving a compression therapy or therapeutic modality compared to a control/comparison intervention.
3. Reported an objective measure for lymphedema (e.g., circumference measurement, bioimpedance spectroscopy or limb volume) of the upper limb or region (e.g., arm, chest wall or breast), lower limb or region (e.g., leg, genital region, or pelvis), or head and neck region.

We excluded reviews or trials that exclusively focused on primary lymphedema; involved children and adolescents; and examined one-time interventions (i.e. a single treatment session only).

### Study selection

Pairs of reviewers (MLM, MMA, SMS) worked independently to identify eligible SRs and RCTs by screening titles, abstracts, and then full texts. The published paper of eligible RCTs identified in a SR were obtained for full text screening and review. We used a structured protocol to ensure that we obtained the most relevant information for each RCT. At each stage, disagreements were resolved by consensus or arbitrated by the third reviewer, if necessary.

### Data extraction for mapping review

The primary outcomes of interest for the mapping review were lymphedema volume and incidence. Data relevant to limb volume or incidence were abstracted from eligible RCTs that were either identified in a SR or a recently published RCT (i.e., not included in a SR). Only the RCTs reporting data on lymphedema incidence or volume were included in the mapping process. The limb volume outcome was assessed as a dichotomous variable for prevention trials (i.e., the proportion of participants who developed lymphedema) or as continuous outcome for RCTs examining interventions (i.e. change in limb volume after the intervention).

Interventions were grouped by the phase of lymphedema treatment, type of intervention and body region of interest (Table 1). The phases of treatment included the prevention phase, intensive reduction phase and maintenance phase. Within the intensive reduction phase, trials were further sub-classified based on whether the compression therapy or therapeutic modality was:

1. An alternative intervention to a component of the CDT regimen (i.e., the experimental intervention replaced a component of the CDT regimen);
2. An adjunctive intervention to the CDT regimen (i.e., the experimental intervention was added to the CDT regimen);
3. A stand-alone reduction phase intervention.

**Table 1:**
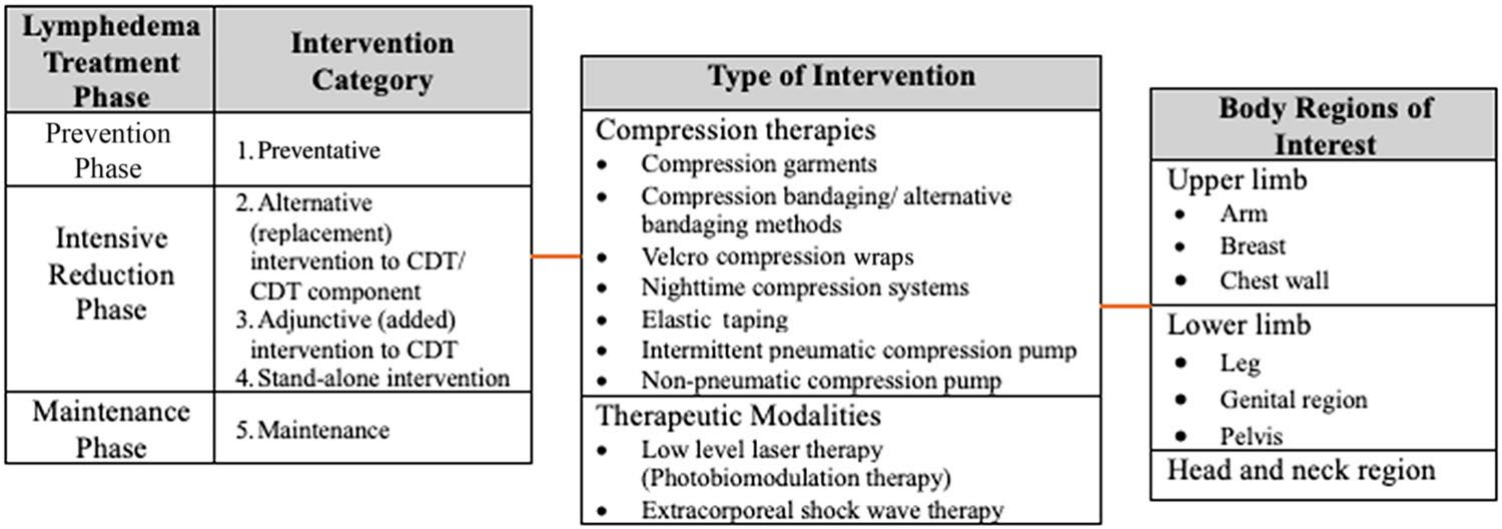
Categories for trials of interest for review

### Risk of *bias* (quality) assessment

Pairs of reviewers (MLM, MMA, SMS) independently reviewed study quality and risk of bias. Discrepancies were resolved by the third reviewer as necessary. The risk of bias of RCTs was appraised using the Cochrane risk-of-bias tool (version 2) [16]. A summary of the risk of bias domains was produced for each of the five intervention categories within the three phases of lymphedema treatment listed in Table 1. A ‘low risk of bias’ indicates the study reporting is adequate for a quality domain and that no major or minor sources of bias are likely to influence results [16]. However, a ‘high risk of bias’ indicates that there are serious errors in study conduct or reporting for the domain, or there are large amounts of missing information. An ‘unclear risk of bias’ is assigned if there is inadequate information in the publication to assess bias for the respective domain [16].

### Data synthesis and analysis

Statistical analyses were conducted using a specialized statistical package (IBM Corp. Released 2024. IBM SPSS Statistics for Windows, Version 29.0.1.0 Armonk, NY: IBM Corp). We calculated the intervention effect for each individual RCT using the change in limb volume for both the intervention and control/comparison group using forest plots. We did not pool the results of trials in the forest plots due to high heterogeneity across studies in population characteristics, outcome measurement methods and timing, and chosen experimental interventions and comparison/control interventions. Instead, we constructed evidence maps for each category using the between group intervention effect for each trial. We created the evidence map using six dimensions:

1. The direction of effect was depicted along the X-axis (i.e., left side: favoring the comparison/control group; middle: insignificant; or right side: favoring the intervention group).
2. The total number of participants in each trial was represented by the Y-axis.
3. The size of each bubble was directly proportional to the between group estimated effect size.
4. The bubble color was represented by the respective intervention type.
5. Within each bubble the solid color pattern represented upper limb or region and the dashed pattern represented the lower limb or region.
6. The risk of bias of the trial was represented by the color of the outline of the bubble in the figure (i.e., red = high risk, yellow = unclear risk, green = low risk).

## Result

**Fig. 1:**
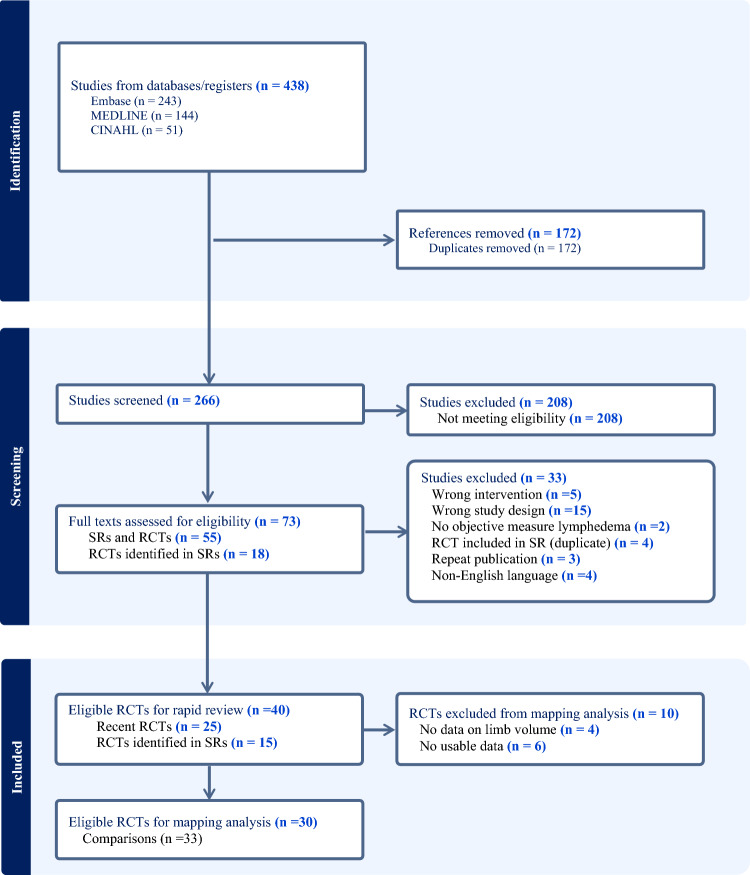
Summary of trial selection process for mapping

The electronic search yielded 438 potentially relevant citations (Fig. 1). Ten systematic reviews and 26 RCTs resulted in the identification of 40 eligible RCTs enrolling 2841 participants [12, 17–55]. Data from 30 RCTs with 33 comparisons (data from one RCT was included in two categories [33]) presenting data on lymphedema volume or incidence were included in the mapping process [12, 17–29, 31, 33, 34, 36, 37, 40–44, 49–53, 55]. The most studied cancer group was breast cancer (37 studies); and the most studied interventions across categories were compression therapies (16 trials), elastic taping (12 trials), and compression pumps (six trials). Further details on the included RCTs are provided in Table 2.

**Table 2:** RCTs of interventions across the treatment phases of lymphedema

Study/Country/ Cancer Type	Population/LE definition	Preventative intervention	Comparison intervention	Timelines & measurement method	Reported results
Secondary Prevention: upper limb (prior to onset of lymphedema)					
Nadal Castells, [20] Spain Upper limb	N = 70 with BCRL undergoing ALND LE incidence: > 200mls or > 10% difference between limbs	n = 35 Age: 58.9 (12.7) 1-h education session + 12-week exercise session + CG (8-h per day for 3 months; 2-h per day after)	n = 35 Age: 56.1 (12.7) 1-h education session + 12-week exercise program	End of 12-week exercise program, 6 months, 1 year and 2 years Limb volume calculated from circumference measurements	2-year follow-up: no significant difference in incidence between groups (12.5% CG group vs. 12.1% comparison group)
Ochalek, [21] Poland Upper limb	N = 41 with BCRL undergoing ALND LE incidence: > 10% difference from pre-operative values	n = 20 Age: 52.9 (9.3) Exercise + circular-knit class I (15–21 mmHg) CG – replaced every 6 months	n = 21 Age: 64.0 (8.6) Exercise alone	1 year and 2 years Limb volume calculated from circumference measurements	1-year follow-up: no significant difference between groups (3 of 20 in CG group compared to 6 in the comparison group)
Paskett, [23] United States Upper limb	N = 554 females with BCRL LE incidence: ≥ 10% increase in affected limb volume	n = 312 Age: 58 (27–88) Education + exercise prescription CG (20–30 mmHg) to wear during exercise, air travel or vigorous activities	n = 242 Age: 59 (24–83) Education alone	Pre-operatively, 6-months, 1 year, and 18 months Limb volume calculated from circumference measurements	18-month follow-up: no significant difference between group: (140 of 312 in CG group vs 101 of 242 in comparison group)
Paramanandam, [22] India Upper limb	N = 301 females with BCRL LE incidence: ≥ 10% difference between limbs/ ≥ 10% change from baseline	n = 152 Age: 46.7 (10.4) Education + CG (20–25 mmHg) – worn post-op day 1 to 3 months post adjuvant cancer treatments: 8 h per day	n = 149 Age: 47.0 (11.7) Education alone	Pre-operatively, 2–4 weeks post-surgery, 5–7 months and at 1 year Limb volume calculated from circumference measurements	1-year follow-up: significantly reduced incidence in favor of CG group (22 of 152 in CG group compared vs 37 of 149 in comparison group)
Tertiary prevention: upper limb (at time of early presentation of lymphedema)					
Blom, [17] Sweden Upper limb	N = 75 females with mild BCRL ≥ 2% increase LE incidence: ≥ 10% difference between limbs	n = 33 Age: 57.9 (13.8) Self-care: counseling exercise, weight control, skin care and self-massage + CG (Class 1 or adjusted Class 2)	n = 37 Age: 57.0 (12.5) Self-care alone	Post-operatively at 1 month, 2, 3 and 6 months Limb volume: water displacement	6-month follow-up: no significant difference between groups (4 of 33 in CG group vs 10 of 37 in comparison group)
Bundred, [18] United Kingdom Upper limb	N = 143 females with BCRL LE incidence: arm volume increase > 10% from baseline	n = 69 Age: 55.8 (32, 86.9) Education, elevation, exercise, and self-massage + CG (20–24 mmHG)	n = 74 Age: 55.5 (33.5, 89.9) Education, elevation, exercise, and self-massage	Pre-operatively, then at 1, 3, 6. 9, 12, 18 and 24 months Limb volume: perometry	2-year follow-up: no significant difference between groups (18 of 61in CG group vs 25 of 61 in comparison group)
Secondary prevention: lower limb					
Hnin, [19] Singapore Lower limb	N = 56 females with gynecologic cancer Unilateral or bilateral LE incidence: Percentage change of > 15% or physical exam	n = 26 Age: 49.6 (24–66) Education + Custom CG (14–21 mmHg) worn: minimum 6 weeks, h worn reported	n = 30 Age: 47.8 (27–66) Education alone	Every 3 months in first year, every 4 months in second year Limb volume: perometry	2-year follow-up: no significant difference between groups (2 of 26 in CG vs 4 of 30 in comparison group)
Wang, [25] China Lower limb	N = 117 females with cervical cancer at high risk of LE LE incidence: clinical examination, symptoms + > 2% excess	n = 59 Age: 46.6 (9.3) Health education + modified CDT: self-MLD, aerobic exercise, + CG (15–30 mmHg)	n = 58 Age: 48.4 (9.8) Health education alone	Within 7–10 days after surgery, then 1, 3, 6 and 12 months Limb volume calculated from circumference measurements	Significant benefit in favor of CG (8 of 58 in modified CDT group vs 20 of 59 in comparison group)
Shallwani, [24] Canada Lower limb	N = 36 females with gynecologic cancer LE incidence: clinical examination	n = 18 Age: 56.3 (10.1) Education and aerobic and resistance exercise prescription + standard or custom CG (18–21 mmHg) to be worn 12–16 h/day for 6 months	n = 18 Age: 58.9 (9.1) Education and standard care (CG provided if LE developed)	Pre-operatively, 4–6 weeks post-op, then at 3, 6 and 12-months Limb volume calculated from circumference measurements and perometry	No significant difference. 1-year follow-up (5 of 18 in CG group vs 5 of 18 in comparison group)
Alternative intervention to compression bandaging					
Oh, [31] Korea Upper limb	N = 42 patients with BCRL	N = 21 Age: 57.3 ± 5.6 yrs CDT using spiral method for CB; 2-week intervention	N = 21 Age: 56.4 ± 7.9 yrs CDT using spica/figure-of-eight method for CB; 2-week intervention	Baseline, post-treatment (2 weeks) Limb volume: water volumetry: distal and proximal segments	The spica method/significantly better volume reduction than the spiral method
Pujol-Blaya, [34] Spain Upper limb	N = 42 patients with BCRL	N = 22 Age: 58.6 ± 12.1 yrs MLD + precast adjustable compression system; daily × 2 weeks; then 3 days (alternate days) per week × 7–14 days until garment received	N = 20 Age: 60.4 ± 12.1 yrs MLD + CB; daily × 2 weeks; then 3 days (alternate days) per week × 7–14 days until garment received	Baseline, post-treatment (2 weeks) and 3-months follow-up Limb volume: water volumetry Circumference measurements for distal and proximal segments	No significant difference between groups
Dhar, [28] India Upper limb	N = 49 female patients with BCRL	N = 25 Age: 50.8 ± 10.2 yrs Mobiderm reapplied 3 × during week 1 and 1 × during week 2	N = 24 Age: 54.9 ± 11.1 yrs CB reapplied 3 × during week 1 and 1 × during week 2	Baseline, post-treatment (15 days) Limb volume: water volumetry	Significant benefit in favour of Mobiderm over traditional CB
Alternative intervention: extracorporeal shock wave therapy (ESWT)					
Cebicci, [27] Turkey Upper limb	N = 20 female patients with BCRL	N = 10 Age: 51.6 ± 6.6 yrs ESWT; 3 sessions/week × 12 sessions	N = 10 Age: 57.9 ± 6.9 yrs CDT; 5x/week for 20 sessions	Baseline, post-treatment (4 weeks) Limb volume: water displacement Circumference measurements	No between group differences
Alternative intervention: compression pump (CP)					
Haghighat, [29] Iran Upper limb	N = 112 unilateral BCRL LE: ≥ 10% difference in limbs	n = 56 Age: 52.7 (10.8) years Modified CDT (limited MLD) + CP for 30 min @ 40 mmHg pressure: daily for 5 days per week for 2–3 weeks Maintenance: CG, night CB, exercise, and self-massage	n = 56 Age: 53.4 (11.4) years CDT: daily 5 days per week for 2–3 weeks Maintenance: CG, night CB, exercise, and self-massage	Baseline, post-treatment (2–3 weeks), 3-month follow-up Limb volume: water displacement	Significant benefit in favor comparison intervention post-intervention No significant difference between groups at 3-month follow-up
Sanal-Toprak, [35] Turkey Upper limb	N = 46 patients with advanced-stage BCRL	N = 22 Age: 55.3 ± 10.3 yrs Pneumatic compression & CB; 3 days/week × 5 weeks	N = 24 Age: 59.0 ± 2.8 yrs MLD and CB; 3 days/week × 5 weeks	Baseline, post-treatment (5 weeks), and 3-month follow-up Circumference measurements	No reported limb volume data There were no significant between group differences
Alternative intervention: elastic taping (ET)					
Smylka, [37] Poland Upper limb	N = 43 females with BCRL LE: Stage II or III with > 20% difference	n = 20 Age: 67.3 (12.0) years IPC, MLD and ET: 3x/ week for 4 weeks n = 22 Age: 65.4 (13.1) years IPC, MLD and sham ET: 3x/ week for 4 weeks	n = 23 Age: 66.5 (12.0) years IPC, MLD, CB: 3 × per week for 4 weeks	Baseline, post-intervention (4 weeks) Limb volume: perometry	Analysis of ET and CB intervention groups only No significant differences between groups post-intervention
Pekyavas, included in alternative and adjunctive categories [33] Turkey Upper limb	N = 30 BCRL LE: Stage II or III LE	n = 15 Age: 58.0 (8.5) years CDT: daily, 10-day treatment with ET replacing the CB Maintenance: self-massage, exercise, and CG	n = 15 Age: 49.6 (10.5) years CDT: daily, 10-day treatment period Maintenance: self-massage, exercise, and CG	Baseline, post-intervention (Day 10), and 1-month post-intervention follow-up Limb volume calculated from circumference measurements	Analysis of ET and CB intervention groups only No significant differences between groups post-intervention
Melgaard, [30] Denmark Upper limb	N = 10 females with Level 2 BCRL	N = 5 Age: 63.0 ± 9.8 yrs CDT with ET: 2 days/week × 4 weeks	N = 5 Age: 62.0 ± 5.8 yrs CDT with CB; daily × 5 days/week × 4 weeks	Baseline and post-treatment (4 weeks) Circumference measurements	No reported limb volume data Significant benefit in favor of the ET group post-intervention
Ozsoy-Unubol, [32] Turkey Upper Limb	N = 35 patients with early-stage BCRL	N = 16 Age: 50.6 ± 6.5 yrs ET: 3 4-day intervals × 4 weeks	N = 19 Age: 54.5 ± 7.5 yrs Compression garment: 23 h/day × 4 weeks	Baseline, post-treatment (4 weeks), and 3-month follow-up Circumference measurements	No reported limb volume data
Tantawy, [38] Egypt Upper limb	N = 59 female patients with BCRL	N = 30 Age: 54.3 ± 4.2 yrs ET: 2x/week × 3 weeks	N = 29 Age: 55.2 ± 3.3 yrs Compression garment: daily × 15–18 h × 3 weeks	Baseline and post-treatment (3 weeks) Circumference measurements	No reported limb volume data Analysis of post-intervention scores favor ET group*
Basoglu, [26] Turkey Upper limb	N = 36 female patients with Level 2 BCRL	N = 17 Age: 53.7 ± 8.6 yrs ET: 1x/week × 4 weeks	N = 19 Age: 53.4 ± 8.3 yrs CDT; 1x/week × 4 weeks	Baseline, post-treatment (4 weeks), and 1-month follow-up Limb volume calculated from circumference measurements	Significant benefit in favor of CDT group post-intervention
Alternative: multiple comparison interventions					
Torres-Lacomba, [39] Spain Upper limb	N = 146 female patients with Stage I and II BCRL Age: 58.4 ± 11.4 yrs	N = 118 Intervention details: simplified CB, MLD, exercise, and pneumatic compression (group 1); cohesive CB, MLD, exercise, and pneumatic compression (group 2); adhesive CB, MLD, exercise, and pneumatic compression (group 3); ET, MLD, exercise, and pneumatic compression (group 4; daily × 5 days/week × 2 weeks for intensive phase; then alternate days × 1 week	N = 28 Intervention details: multilayer CB, MLD, exercise, and pneumatic compression; daily × 5 days/week × 2 weeks for intensive phase; then alternate days × 1 week	Baseline, post-intervention (3 weeks) Limb volume calculated from circumference measurements	Limb volume data presented as median and range Simplified CB more effective than traditional CB: Traditional CB more effective than Adhesive CB; No difference: Cohesive; Traditional CB more effective than ET
Selcuk Ylimaz, [36] Turkey Upper limb	N = 48 patients with mild Stage 2 BCRL	N = 33 Age: 51.4 ± 10.7 yrs (ET group); 55.3 ± 12.1 yrs (LLLT group) ET and CB (ET group) or LLLT and CB (LLLT group): 15 sessions over 3 weeks, followed by maintenance with CG	N = 15 Age: 57.6 ± 9.5 yrs MLD (Vodder massage × 30–45 min/ session) and CB (MLD group): 15 sessions over 3 weeks, followed by maintenance with CG	Baseline, post-intervention (3 weeks), then 4 and 12 weeks after end of treatment Limb volume calculated from circumference measurements	Significant difference in favor of the ET group compared to the MLD group post-treatment
Adjunctive intervention: extracorporeal shock wave therapy					
El-Shazly, [40] Egypt Upper limb	N = 60 females BCRL (age range of 30 to 50 years) LE definition: unilateral; Stage II or III (advanced stage)	n = 30 Age: not reported MLD, exercises, shoulder ROM, IPC: 3x/ week for 6 weeks + ESWT: 2x/ week for 6 weeks 2022	n = 30 Age: not reported MLD, exercises, shoulder ROM, IPC: 3x/ week for 6 weeks	Baseline and post-intervention (6 weeks) Limb volume: method not reported	Significant benefit in favor of ESWT
Mahran, [41] Egypt Upper limb	N = 40 post-menopausal females with BCRL LE: 2 cm to 8 cm difference in circumference at any point or > 200 ml difference in limbs	n = 20 Age: 52.1 (4.0) years CDT 3x/week for 8 weeks + ESWT 2x/ week for 8 weeks	n = 20 Age: 53.8 (3.4) years CDT 3x/week for 8 weeks	Baseline, post-intervention (4 weeks) and 8 weeks Limb volume: water displacement	Significant difference in favor of the ESWT at 4 weeks and 8 weeks
Lee, [12] Korea Upper limb	N = 28 females with BCRL LE: Stage II; > 2 cm between arms at points on arm, and a volume difference of 200 + mls	n = 14 Age: 57.5 (11.2) years CDT daily for 3 weeks + ESWT 3x/ week for 3 weeks	n = 14 Age: 53.2 (8.6) years CDT daily for 3 weeks	Baseline, post-treatment (3 weeks), and 3 months Limb volume: water displacement	No significant differences between the groups at the 3-week or 3-month follow-up
Adjunctive intervention: compression pump					
Szuba, [44] United States Upper limb	N = 23 females with BCRL LE: ≥ 20% difference in limbs	n = 12 Age: 68.8 (9.1) years CDT + IPC × 30 min @ 40–50 mmHg pressure: daily for 2 weeks	n = 11 Age: 65.0 (10.8) years CDT: daily for 2 weeks	Baseline, post-intervention (10 day), 1-month follow-up Limb volume: water displacement	Significant benefit in favor of IPC post-intervention*
Szolnoky, [43] Hungary Upper limb	N = 27 females with unilateral BCRL LE: definition not reported	n = 14 Age: mean 56.6 years CDT with MLD × 30 min + IPC × 30 min: daily 5 days per week for 2 weeks	n = 13 Age: mean 54.8 years CDT (MLD: 60 min): daily 5 days/ week for 2 weeks	Baseline, start of intervention, post-intervention, 1 and 2-month follow-ups Limb volume calculated from circumference measurements	Significant benefit in favor of IPC at all time points*
Adjunctive intervention: low level laser therapy (LLLT)					
Mogahed, [42] Egypt Upper limb	N = 30 females with BCRL LE: Stage II and III	n = 15 Age: 48.4 (4.1) years MLD, exercise, IPC: 3x/ week for 12 weeks + LLLT: infrared, pulsed; axillary region; 2 Joules/cm^2^	n = 15 Age: 48.3 (4.1) years MLD, exercise, IPC + Placebo LLLT	Baseline, post-intervention Limb volume: water volumetry	Significant difference in favor of LLLT
Adjunctive intervention: elastic taping (ET)					
Pekyavas,—included in alternative and adjunctive categories [33] Turkey Upper limb	N = 30 BCRL LE: Stage II or III LE	n = 15 Age: 58.0 (8.5) years CDT: daily, 10-day treatment with ET applied under CB Maintenance: self-massage, exercise, and CG	n = 15 Age: 49.6 (10.5) years CDT: daily, 10-day treatment period Maintenance: self-massage, exercise, and CG	Baseline, post-intervention (Day 10), and 1-month post-intervention follow-up Limb volume calculated from circumference measurements	Analysis of two of three intervention groups No significant differences between groups post-intervention
Collins, [46] Ireland Breast edema	N = 14 females with BCRL LE: a ratio of ≥ 1.1:1 tissue water difference	n = 7 Age: 64.1 (5.9) years MLD × 20 min, 1x/ week for 3 weeks + ET for two 7-day periods	n = 7 Age: 53.9 (10.4) years MLD × 20 min, 1x/ week for 3 weeks	Baseline, post-intervention (2 weeks), and 6-week post-intervention follow-up Breast percentage tissue water	Measures of variability not reported No between group analyses reported
Atar, [45] Turkey Head & Neck	N = 58 with HNL Males: 35; Females: 23 LE: edema ≥ 3 months; excluded MDACC HNL scale levels 0 and 3	n = 30 Age: 51.5 (7.7) years Daily MLD × 1 week, then MLD 2 × per week for 3 weeks; daily exercise × 4 weeks; ET applied daily × 1 week, then 3x/ week for 3 weeks. Maintenance: daily home exercises week 5–8	n = 28 Age: 51.1 (8.8) years Daily MLD × 1 week, then MLD 2 × per week for 3 weeks; daily exercise × 4 weeks; sham ET applied daily × 1 week, then 3x/ week for 3 weeks. Maintenance: daily home exercises week 5–8	Baseline, post-intervention and 8-week follow-up Tape measurements for external LE; Fiber endoscopic images for internal LE analyzed into categories	Data analyzed for total circumference A significant benefit found in favor of ET group for external LE
Stand-alone intervention: low level laser therapy (LLLT)					
Carati, [48] Australia Upper limb	N = 61 females with BCRL LE: > 200 ml difference or ≥ 2 cm difference circumference at 3 points	n = 33 Age: 65 (2) years LLLT: 9 sessions (3x/ week for 3 weeks) with 17 treatment points (17 min) @ dosage of 1.5 Joules/cm^2^	n = 28 Age: 63 (2) years Sham LLLT: 9 sessions (3x/ week for 3 weeks) with 17 treatment points—no laser delivered	Baseline, post-intervention (3 weeks), 1-month, and 2–3-month post-intervention follow-up Limb volume: perometry	No data to allow for analysis prior to cross-over Significant benefit in favor of LLLT at follow-up
Lau, [50] China Upper limb	N = 21 females with BCRL LE: > 200 mL difference	n = 11 Age: 50.9 (8.6) years LLLT: 12 sessions—3x/ week for 20 min for 4 weeks; 50 cm laser head for 144cm^2^ area; @ dosage 2 Joules/cm^2^	n = 10 Age: 51.3 (8.9) years Control: no intervention	Baseline, post-intervention (4 weeks), 4-weeks post-intervention follow-up Limb volume: water volumetry	No significant difference between the groups post-intervention; Significant benefit in favor of laser therapy at follow-up
Storz, [54] Germany Upper limb	N = 36 females with BCRL LE: 3-month history of LE	n = 17 Age: 61.1 (9.7) years LLLT: 8 sessions—2x/ week for 4 weeks for 10 min; 4.9 cm^2^ treatment head; 78.54 cm^2^ treatment area @ dosage of 4.89 Joules/cm^2^	n = 19 Age: 59.4 (10.2) years Placebo LLLT	Baseline, post-intervention (4 weeks), 4, 8, 12 weeks post-intervention follow-up Limb volume calculated from circumference measurements	No data on mean or standard deviation No significant differences between the groups post-intervention or at any follow-up
Stand-alone intervention: elastic taping (ET)					
Malicka, [51] Poland Upper limb	N = 28 females with early-stage BCRL LE: Grade 1 lymphedema	N = 14 60.1 (6.3) Intervention details: ET (two subgroups: 1) upper extremity with single fan shapes; 2) upper extremity in a double fan shape with arm and forearm); applied 1x/week × 4 weeks	N = 14 59.5 (5.7) Intervention details: no treatment	Baseline, 2 weeks, and post-intervention (4 weeks) Limb volume calculated from circumference measurements	Within-group difference for ET group; between-group differences not presented
Stand-alone mixed interventions					
Abdelhalim, [47] Egypt Upper limb	N = 43 females with BCRL LE: difference of 2 to 8 cm at a single measurement site between arms	n = 21 Age: 48.7 (3.1) ESWT 2500 shocks per session @ frequency of 4 Hz and flow density of 90 mJ; 3x/ week for 4 weeks (12 sessions) Daily home program: range of motion, pumping exercises and elevation	n = 22 Age: 49.6 (2.8) IPC (60 mmHg) for 45 min, 5 times/week for 4 weeks (20 sessions) Daily home program: range of motion, pumping exercises and elevation	Baseline and post-intervention (4 weeks) Circumference measurements	No data on limb volume Significant difference between groups at 4 measurement levels favouring ESWT group
Maintenance: night-time compression					
Mestre, [53] France Upper Limb	N = 40 females with BCRL post intensive CDT LE: eligible if a > 10% reduction in Phase I CDT	n = 20; Age: 65.1 (8.6) Daytime CG (Class I or II) + Night-time Compression system	n = 20; Age: 68.9 (11.8) Daytime CG (Class I or II)	Baseline, then Day 30 and Day 90 (fast-track Day 30 to 90) Limb volume calculated from circumference measurements	At Day 30 post-intervention: no significant differences between groups
McNeely, [52] Canada Upper limb	N = 120 females with BCRL LE: ≥ 200 ml or ≥ 10% difference between limbs	n = 44; Age: 62 (9) Education, Daytime CG (Class II) + Night-time CB Group: 8 h/ night for 5 nights x 6wks, then 3 nights × 6 wks n = 37; Age: 62 (12) Education, Daytime CG (Class II) + Night-time Compression System Group: 8 h for 5 nights/wk for 6wks, then 8 h × 3 nights/wk for 6 wks	n = 39; Age: 59 (11) Standard care: Education + Day-time CG (Class II)	Baseline and then 6, 12, 18, 24 weeks (fast-track from 12–24 weeks) Limb volume: perometry	At 12-weeks post-intervention: significant benefit in favour of NCSG and CB groups vs comparison group
Maintenance: compression pump					
Fife, [49] United States Upper Limb	N = 36 LE: eligible if ≥ 5% excess volume	n = 18; Age: 63.9 (12.2) Advanced pneumatic compression device including arm, adjacent chest, and truncal quadrant: home treatment 60 min/ day for 12-weeks @ a pressure between 9 and 13 mmHg	n = 18; Age: 59.7 (12.6) Standard pneumatic compression device with 4 chambers for arm: home treatment 60 min /day for 12-weeks @ pressure of 30 mmHg	Baseline, and then 12-weeks post-intervention Limb volume calculated from circumference measurements	At 12-weeks post-intervention: significant benefit in favor of Advanced Pneumatic Compression
Rockson, [55] United States Upper Limb	N = 50 females with BCRL LE: diagnosis of upper limb lymphedema	n = 23; Age: 60.5 (10.8) CG + Non-pneumatic wearable compression system for a minimum of 60 min/ day for 28 days	n = 27; Age: 60.3 (10.8) CG + Advanced pneumatic compression for a minimum of 60 min/ day for 28 days	Baseline, then Day 28 post-intervention Limb volume calculated from circumference measurements	At Day 28: analyses based on published data suggest borderline significance

*Age* mean years and standard deviation/range, *ALND* axillary lymph node dissection, *BCRL* breast cancer-related lymphedema, *CB* multicomponent compression bandaging, *CDT* complete decongestive therapy, *CG* compression garment, *ESWT* extracorporeal shockwave therapy, *HNL* head and neck cancer lymphedema, *ET* Elastic Taping, *LE* lymphedema, *LLLT* low level laser therapy, *MLD* manual lymph drainage, *MDACC HNL* MD Anderson Cancer Centre Head and Neck Lymphedema Scale

*Measures of variability from Li, et al. (2022)

### Risk of *bias*

**Fig. 2:**
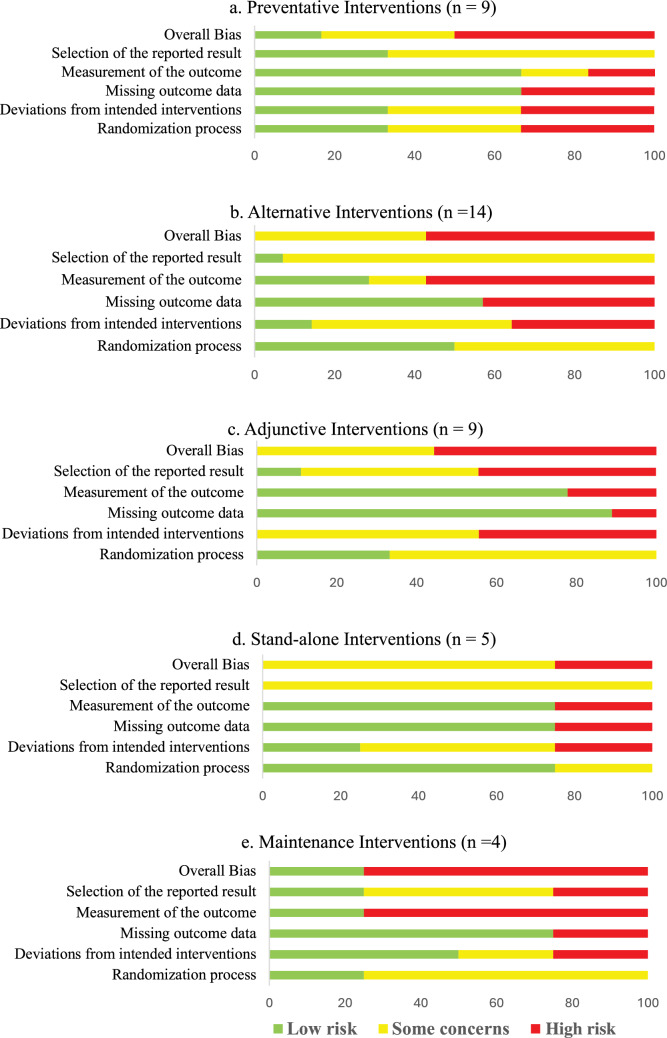
Risk of bias of included trials (N = 40 trials; 41 comparisons)

Figure 2a–e presents the overall risk of bias by review category. Across all categories and domains, most trials were rated as having ‘some concerns’ or a ‘high risk of bias’, especially related to the domains of ‘measurement of the outcome’ and ‘deviations from intended interventions’. Only two trials scored as ‘low risk of bias’, one in the prevention phase [22] and one in maintenance phase [52]. All trials in the CDT intensive reduction phase categories of alternative, adjunctive or stand-alone interventions were scored as having ‘some concerns’ or ‘high risk of bias’ in the overall bias domain.

### Prevention phase

**Fig. 3:**
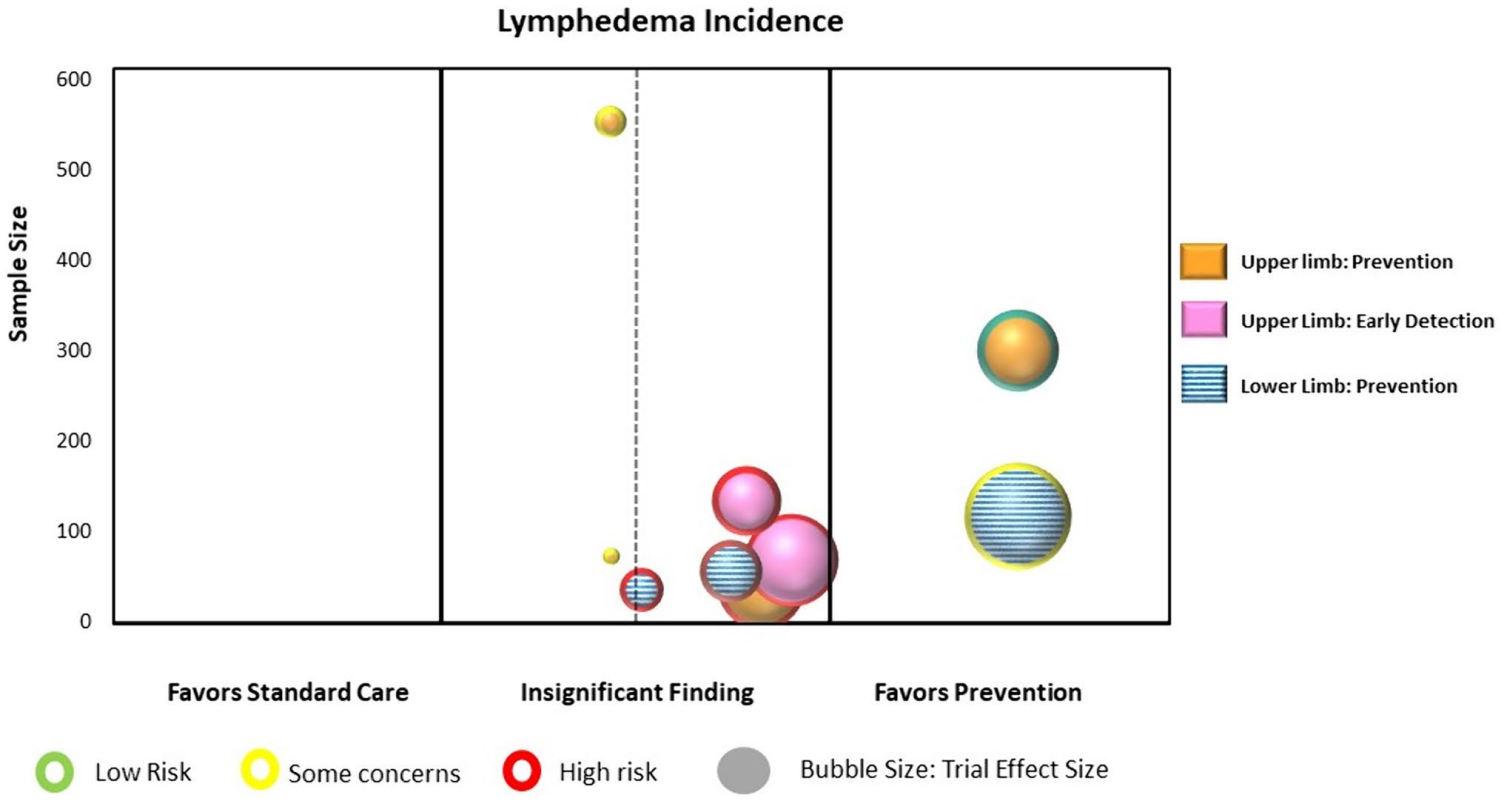
Map of preventative interventions (n = 9 RCTs)

Nine RCTs (1393 participants) were identified that reported on the use of a prophylactic compression garment with or without other interventions for the secondary prevention (i.e., intervention prior to onset of lymphedema) or tertiary prevention (i.e., early detection: intervention at the first presentation of lymphedema) [17–25]. Six trials examined upper limb lymphedema related to breast cancer [17, 18, 20–23], one trial examined lower limb lymphedema in cervical cancer [25], and two trials examined lower limb lymphedema including all gynecologic cancers [19, 24]. Trial sample sizes ranged from 36 to 554 participants (mean 155 participants). All nine trials were included in the analyses and mapping process. Point estimates for six of the nine individual trials favored the use of a prophylactic compression garment with or without other interventions; however, only two trials were found to show a statistically significant difference in favor of compression garment use (Supplementary Material: Fig. 1a) [22, 25]. Figure 3 presents the evidence map.

### Intensive reduction phase: alternative interventions to a CDT component

**Fig. 4:**
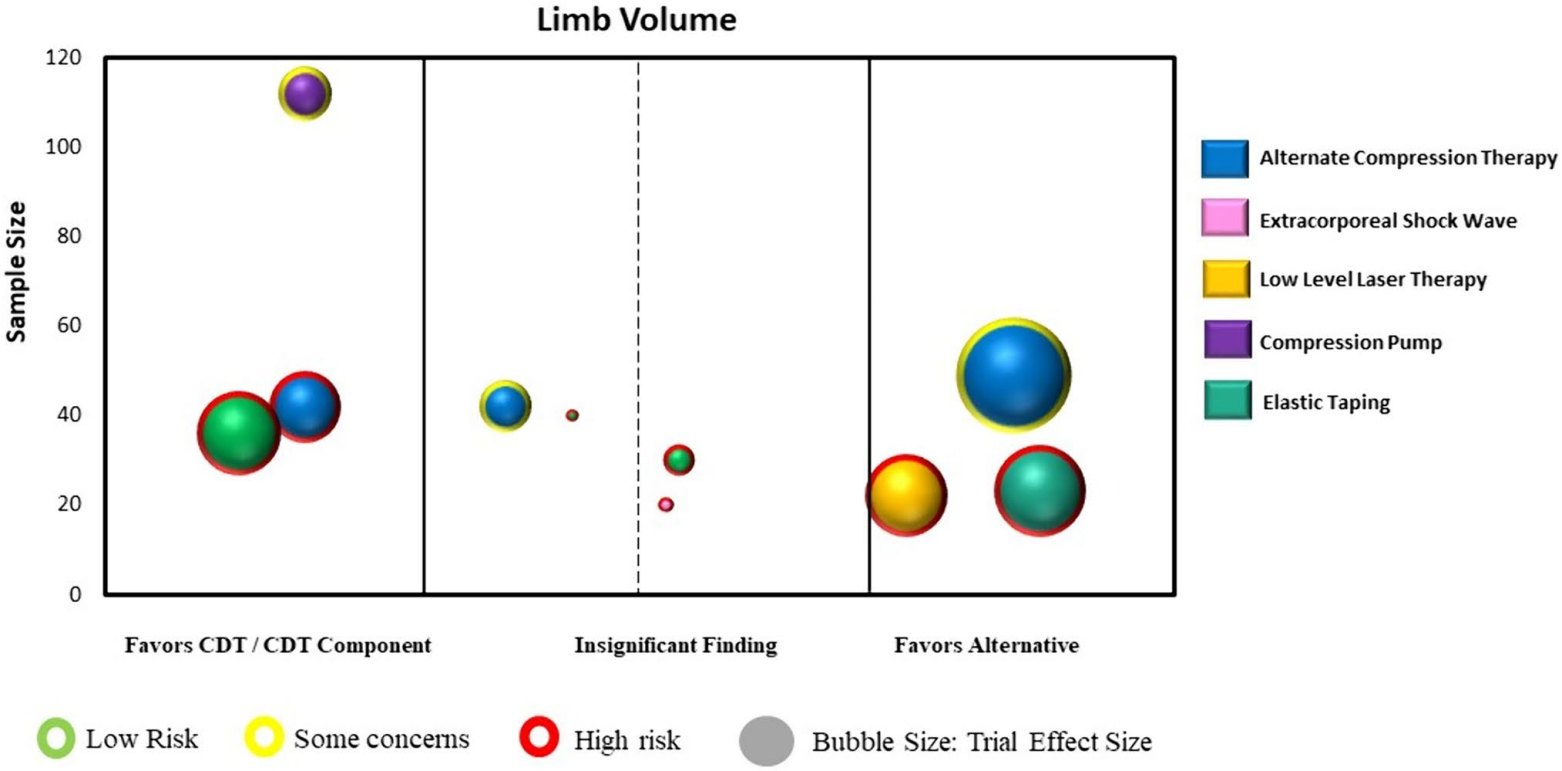
Map of alternative interventions to CDT (n = 9 RCTs with 10 comparisons)

Fourteen RCTs (722 participants) were identified that examined an alternative intervention to CDT or to a component of CDT during the intensive reduction phase [26–39]. All trials involved individuals with BCRL and the measurement of upper limb lymphedema. Three trials examined an alternative compression method to compression bandaging (e.g., spiral bandaging, compression systems) [28, 31, 34], and one trial examined extracorporeal shock wave therapy to CDT [27]. Two trials examined the addition of a compression pump with one trial using the pump along with modified manual lymph drainage massage [29], and the other trial using the pump as a replacement for the manual lymph drainage component [35]. Six trials examined the use of elastic taping as an alternative to compression bandaging [26, 30, 32, 33, 37, 38], while two trials examined multiple alternative interventions including differing types of compression bandaging methods, elastic taping, and low level laser therapy [36, 39]. Trial sample sizes ranged from 10 to 146 (mean 52 participants). Nine trials (10 comparisons) were included in the analysis and mapping process [26–29, 31, 33, 34, 36, 37]. Point estimates fell evenly on both sides of the forest plot; with two trials (three comparisons) showing statistically significant differences favoring the alternative intervention [28, 36] and three trials favoring CDT [26, 29, 31] (Supplementary Material: Fig. 1b). Figure 4 presents the evidence map for alternative interventions.

### Intensive reduction phase: adjunctive interventions to CDT

**Fig. 5:**
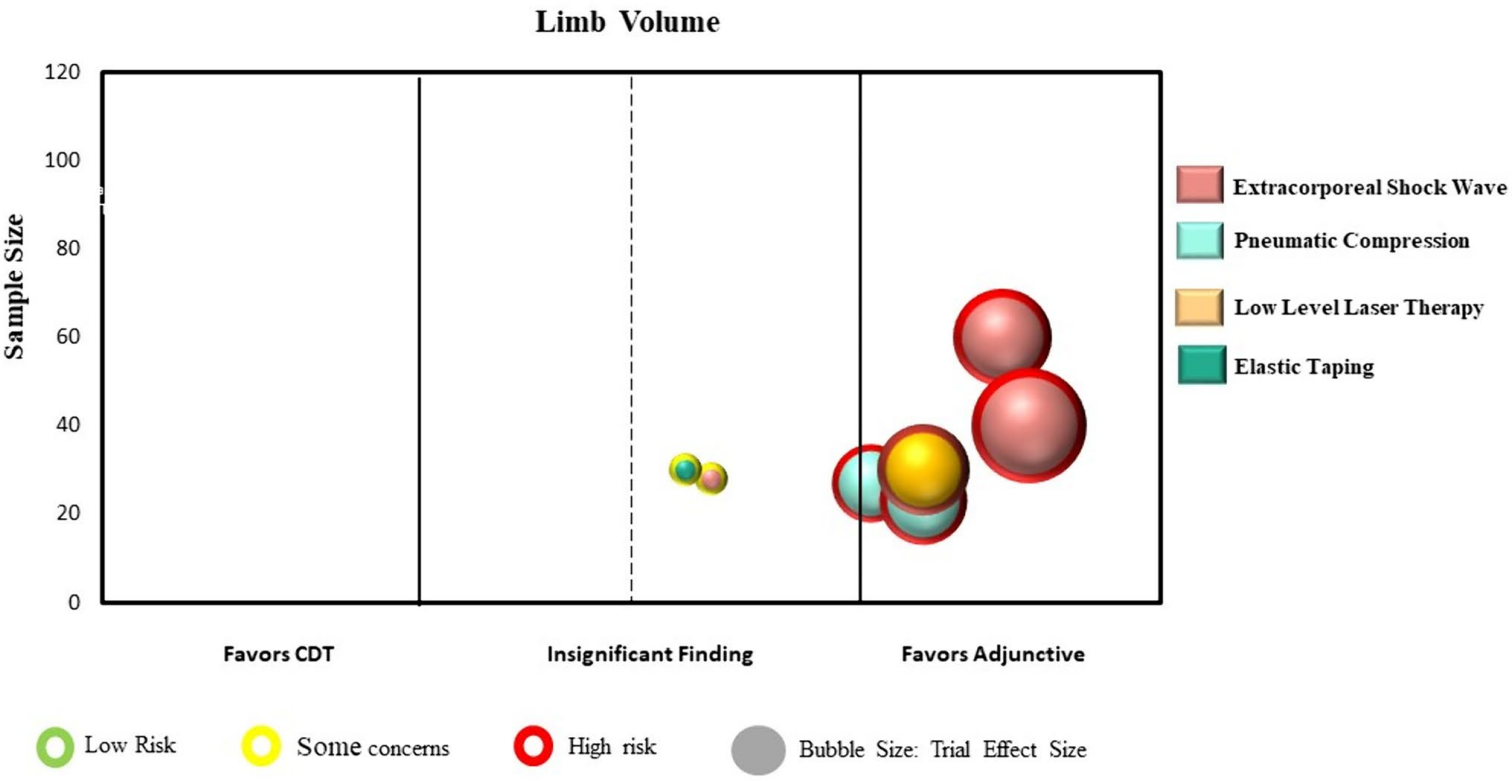
Map of adjunctive interventions to CDT (n = 7 RCTs)

Nine RCTs (310 participants) examined adjunctive interventions to CDT during the intensive reduction phase [12, 33, 40–46]. Eight trials examined individuals with breast cancer, with seven involving the measurement of upper limb volume [12, 33, 40–44] and one including the measurement of breast edema [46]. One trial examined individuals with head and neck cancer with measurement of the face and neck regions [45]. Three trials involved the addition of extracorporeal shock wave therapy [12, 40, 41], two involved the addition of compression pump treatments [43, 44], one involved the addition of low level laser therapy [42], and three involved the addition of elastic taping [33, 45, 46]. Trial sample sizes ranged from 14 to 60 (mean 34 participants). Seven RCTs, all related to breast cancer and upper limb lymphedema, were included in the analysis and mapping process [12, 33, 40–44]. Point estimates for all trials favored the adjunctive intervention to CDT, with four trials showing a statistically significant benefit [40–42, 44] (Supplementary Material: Fig. 1c). Figure 5 presents the evidence map for adjunctive interventions.

### Intensive reduction phase: stand-alone interventions

**Fig. 6:**
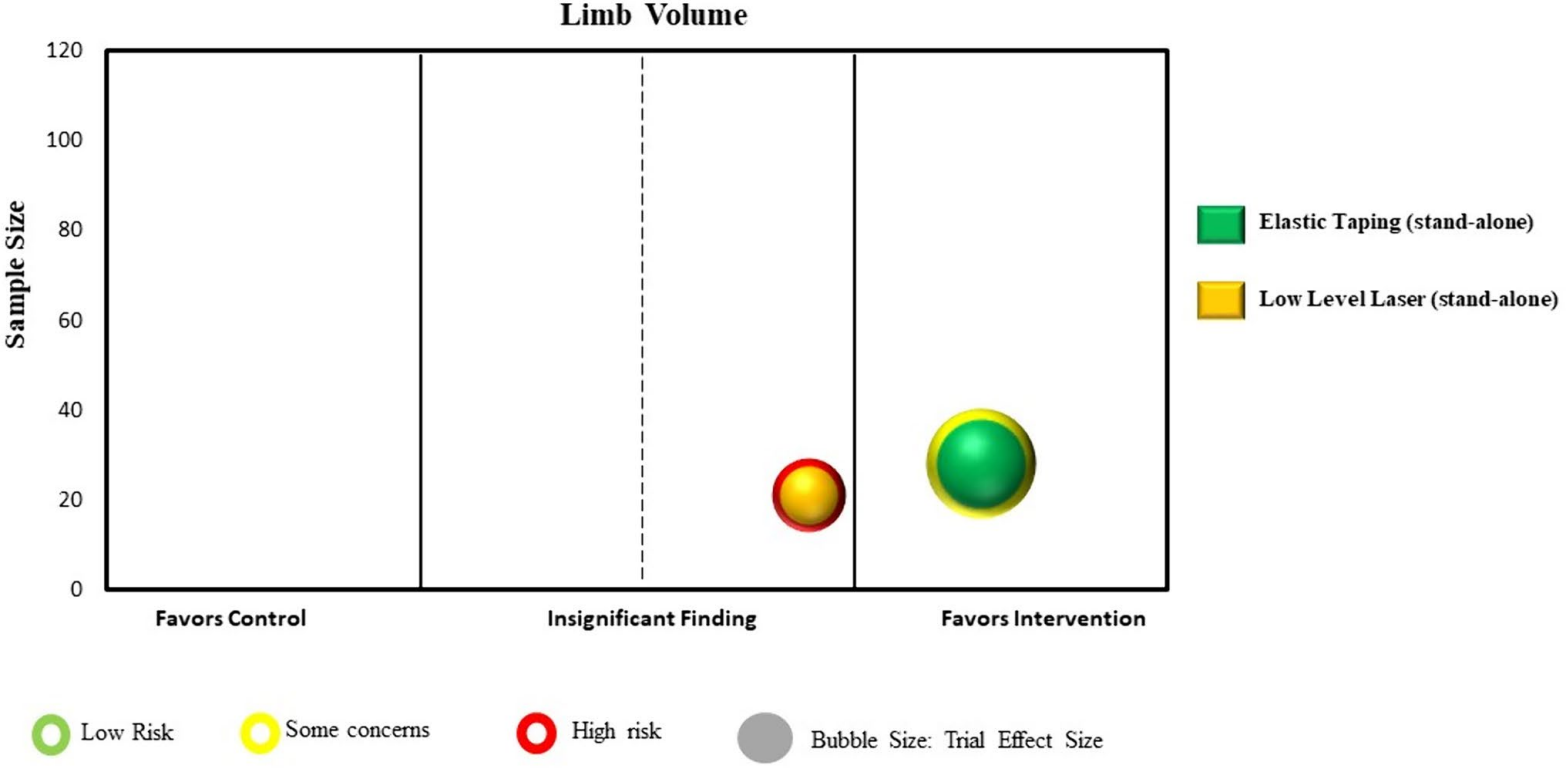
Map of stand-alone interventions (n = 2 RCTs)

Five RCTs (189 participants) examined stand-alone interventions during the intensive reduction phase [47, 48, 50, 51, 54]. All studies examined individuals with breast cancer and performed measurement of upper limb lymphedema. Three RCTs compared low level laser therapy to sham/no treatment [48, 50, 54], one compared elastic taping to no treatment [51], and one compared the effect of extracorporeal shock wave therapy to use of a compression pump [47]. Trial sample sizes ranged from 21 to 61 (mean 38 participants). Two trials were included in the mapping process [50, 51]. Point estimates for both trials favored the stand-alone intervention, with one trial showing a statistically significant benefit [51] (Supplementary Material: Fig. 1d). Figure 6 presents the evidence map for stand-alone interventions.

### Maintenance phase interventions

**Fig. 7:**
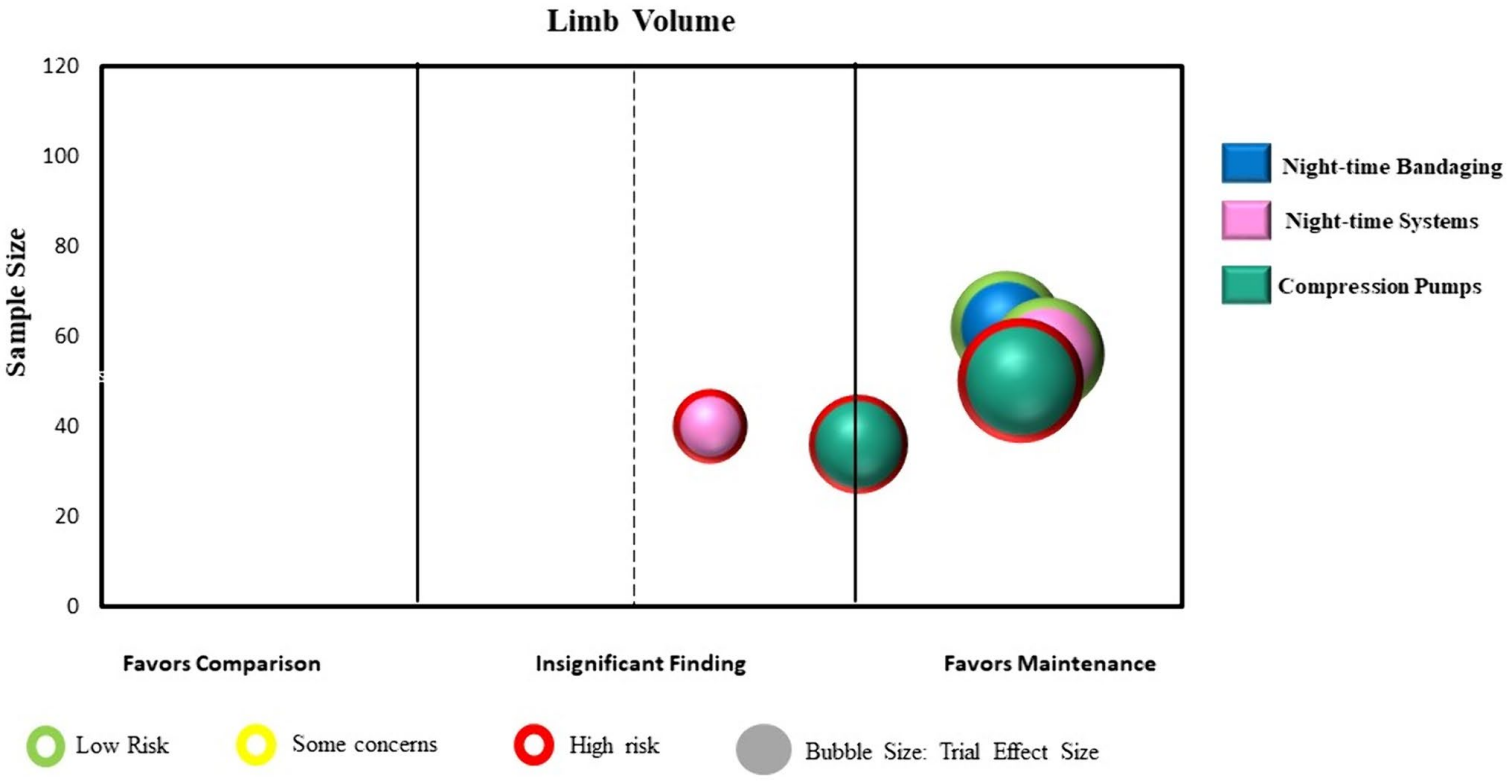
Map of maintenance interventions (n = 4 RCTs with 5 comparisons)

Four RCTs (246 participants) examined interventions in the maintenance phase of lymphedema treatment [49, 52, 53, 55]. All studies examined individuals with breast cancer and performed measurements related to upper limb lymphedema. Two RCTs examined the addition of nighttime compression to daytime use of a compression garment [52, 53], and two RCTs involved the direct comparison of different compression pumps with the newer technology as the experimental intervention [49, 55]. Trial sample sizes ranged from 36 to 120 (mean 62 participants). All four trials, involving five comparisons, were included in the mapping process. Point estimates for all trials favored the maintenance intervention, with three comparisons showing a statistically significant benefit [49, 52] (Supplementary Material: Fig. 1e). Figure 7 presents the evidence map for the maintenance interventions.

### Overall findings

As portrayed in the evidence maps, a paucity of high-quality large-scale trials were found, with only two trials that had both a ‘low risk of bias’ and a sample size of > 100 participants [22, 52]. Ninety-three percent (n = 37) of the trials included participants with a diagnosis of breast cancer, and 90% (n = 36) examined interventions for the upper limb. Most interventions were compared to a comparison intervention rather than no treatment or placebo/sham, making the available evidence less helpful for comparative effectiveness inferences. Moreover, variability was found in the intervention effects from clinically insignificant effects (i.e., small bubble size) to large effects (i.e., large bubble size). Findings from the evidence maps suggest potential for benefit from (1) use of a compression garment for the prevention phase of lymphedema, (2) adjunctive interventions to CDT (i.e., additional interventions added to CDT) in the intensive reduction phase, and (3) the addition of nighttime compression and use of a compression pump in the maintenance phase. Conflicting evidence was found for the use of alternative interventions to standard CDT components in the reduction phase, and limited evidence was found supporting stand-alone interventions in the reduction phase. No studies were found examining stand-alone interventions involving compression garments or compression wraps in the intensive reduction phase.

## Discussion

The present review is the first rapid review with evidence mapping to examine the benefit from compression therapies and therapeutic modalities in the treatment of lymphedema secondary to cancer. Forty studies were mapped to five categories across prevention, intensive reduction, and maintenance phases of lymphedema treatment. A significant evidence gap was identified regarding the methodological quality of trials, especially those conducted in the reduction phase of lymphedema treatment. Twenty-two (55%) of the trials were rated as having ‘a high risk of bias’, and 41% had ‘some concerns’, particularly in the domains of ‘measurement of the outcome’ and ‘deviations in intended outcomes’. Moreover, four trials had unclear statistical methods, or did not report data on point estimates and measures of variability to inform treatment effects. While the level of evidence of RCTs is higher than other study designs, flaws in the trial design, conduct, analyses and reporting can lead to an inaccurate estimation of the intervention effect [16, 56]. Thus, the lack of adequate methodological rigour brings into question the credibility of the individual trial findings [57].

Two trials were scored as ‘low risk of bias’. One trial, conducted in the prevention phase, involved 301 females who had undergone an axillary lymph node dissection for breast cancer. The authors reported a reduced incidence (*p* = *0.03*) and time of onset of upper limb lymphedema (*p* = *0.034*) from wear of a prophylactic compression garment 8 h a day, encompassing the time from day one-to-three post-operatively until 3-months post adjuvant cancer treatment [22]. The other trial (two comparisons) occurred in the maintenance phase. This trial involved a 12-week intervention with 120 females with BCRL that examined the addition of nighttime compression [52]. Benefit was found whether through self-administered compression bandaging (*p* < *0.01*) or use of a nighttime compression system garment (*p* < *0.001*).

Many trial interventions (n = 29; 73%) were administered during the intensive reduction phase of lymphedema treatment. The interest in examining stand-alone as well as alternative and adjunctive interventions to CDT is not surprising. Although CDT remains a fundamental intervention for individuals with lymphedema, the application of components such as manual lymph drainage and multi-layered compression bandaging require therapists with lymphedema-specific certification, limiting access to potentially costly specialized services [2]. Novel self-care solutions such as specially designed compression systems [28, 52] and compression pumps that incorporate mobility [58] may help to improve patient outcomes and reduce costs of care [3]. Moreover, in the opinion of the authors, many of the alternative interventions interfere less with daily activities (e.g., Velcro wraps, elastic taping) than compression bandaging, and are readily available to the patient or practitioner. Further research is needed to optimize protocols for application, and to integrate these alternative interventions into lymphedema management.

A recent Delphi survey identified a total of 12 outcome domains to comprise a core outcome set to capture the burden of lymphedema related to breast cancer [59]. Core outcome sets are standardized and agreed-upon sets of measures that characterize the minimum group of data that should be collected and reported for interventional research for a particular health condition [60]. Importantly, core outcome sets, if agreed upon by a community of stakeholders, ensures that the measures are relevant to the patient and caregiver experience, and to the expertise of clinicians, allied health professionals, and researchers. Use of this recommended core outcome set as a guide for future trials would enable all studies to be compared and combined, and to pre-emptively address reporting bias, wherein some studies, as seen in this mapping review, only report selected outcomes [61].

A particular challenge in the field of lymphedema research is the growing number of published SRs with inconclusive and conflicting findings [11, 13, 62, 63]. The use of a mapping review allowed us to extract data into defined categories, and to provide a visual display of findings to inform the current state of the evidence [64]. Most of the trials included in the review examined interventions for the treatment of lymphedema related to breast cancer, identifying a gap in research for other cancers and body regions impacted by lymphedema. Through this mapping process we were able to better understand the wider context of research in the field and identify a key research gap, namely the paucity of high-quality large-scale trials [64].

### Limitations

Our review was limited by the rapid search method, which may mean some articles were missed. We also limited our mapping process to our primary outcome of limb volume at the post-intervention follow-up time point. In addition, two of the review authors led RCTs that were included in the mapping process; however, neither screened nor evaluated the risk of bias for their respective trial [24, 52]. Despite the limitations, our mapping review provides a visual depiction of the evidence gaps and displays the potential for benefit especially from compression garments for the prevention of BCRL, and from the addition of nighttime compression in the maintenance phase.

## Conclusions

Further high-quality large-scale research is warranted, as findings are likely to change the estimate of effect and associated conclusions of future SRs [65, 66]. Based on these findings, we make the following recommendations for future research involving compression therapies and therapeutic modalities:

1. Further research with respect to populations beyond breast cancer and evaluation of outcomes over the longer-term will address gaps in evidence.
2. Closer attention to trial quality, to reduce the risk of bias, will increase confidence in the findings.
3. Consideration of the recently established lymphedema core outcome set is recommended to enable future studies to be compared and combined.
4. A multi-centre collaborative research approach will support the conduct of large-scale trials to inform the optimal type, timing, and combination of compression therapies and therapeutic modalities as interventions for lymphedema secondary to cancer.

## Supplementary Information

Below is the link to the electronic supplementary material.

Supplementary file1 (DOCX 372 KB)

## Data Availability

No datasets were generated or analysed during the current study.
