# Supplementary material for: The effect of compression therapies and therapeutic modalities on lymphedema secondary to cancer: a rapid review and evidence map
Source: Med Oncol. 2024 Oct 17;41(11):288. doi: 10.1007/s12032-024-02447-w (PMC11486789; doi:10.1007/s12032-024-02447-w)
Supplement: Supplementary file 1 — Supplementary file1 (DOCX 372 KB) [file 12032_2024_2447_MOESM1_ESM.docx]

**Supplementary Material: Appendix A - Search Strategy**

Last 5 years maximum (SR, MA, and RCTs only)

English only

No grey literature (leave out conference abstracts)

1. Compression: compression garments, pressure garments, stockings, lymph* sleeve* or bandag* (around different methods), wrapping or wraps or wrapped or compression wraps, night compression systems, kinesiotaping

2. Devices: laser therap* or light therap* or shock wave therap* or shock* therap* or hifu therap* or (extracorporeal adj5 therap*)

3. Pneumatic compression pumps

**Ovid MEDLINE(R) ALL <1946 to Oct 10, 2023>**

<https://login.ezproxy.library.ualberta.ca/login?url=http://ovidsp.ovid.com/ovidweb.cgi?T=JS&NEWS=N&PAGE=main&SHAREDSEARCHID=6JwhAruxMM24J58WTnE7a9Q4AKahTwv7zmoWVFHQLr78Tj9iUPZrmmcn2CvsKfn7P>

1 breast cancer lymphedema/ 419

2 Lymphedema/ and exp neoplasms/ 3890

3 ((lymphedema or lymphoedema or lymphodema or lymphedoema) and (cancer* or neoplas* or tumour* or tumor* or malignan* or melanoma* or sarcoma* or carcinoma* or metastas* or lymphoma* or axillary dissection* or lymph-node excision*)).mp. 7040

4 1 or 2 or 3 7255

5 bandages/ or compression bandages/ or stockings, compression/ 22363

6 laser therapy/ or low-level light therapy/ 47405

7 Extracorporeal Shockwave Therapy/ 955

8 Intermittent Pneumatic Compression Devices/ 800

9 (compression or bandag* or wrapping or wraps or wrapped or pressure garments or stocking or stockings or lymph* sleeve* or pump or pumps or hose or hosier* or kinesiotap* or laser therap* or light therap* or ((extracorporeal or shock or shockwave or hifu) adj5 (therap* or treatment*))).mp. 409491

10 5 or 6 or 7 or 8 or 9 409491

11 4 and 10 786

12 limit 11 to (english language and yr="2018 -Current") 269

13 exp Clinical trial/ or (randomi* or randomly or (random adj4 (allocat* or distribut* or assign*)) or placebo or trial or groups or subgroups or (phase adj1 ("3" or "2" or "1" or III or II or I))).tw. or rct.ti. 4120305

14 (pubmed or medline or cochrane or scopus or cinahl).tw. or (systematic* adj3 review*).mp. or meta-analy*.pt,mp. or (meta-analy* or meta analy* or metaanalys* or umbrella or research synthesis or overview).tw. or (technology assessment* or hta).mp. or review.pt. 3562327

15 13 or 14 7275659

16 12 and 15 147

17 ((case-control* or (cross-sectional not cross-sectional-area) or cohort or qualitative or (observational adj2 study) or case-series or case-report or case-study or delphi-study or bibliometric-analys* or questionnaire or survey or (tool and validat*)) not (trial or rct or review or metaanal* or meta-anal*)).ti. 888264

18 16 not 17 144

**Embase <1974 to 2023 Oct 10>**

<https://login.ezproxy.library.ualberta.ca/login?url=http://ovidsp.ovid.com/ovidweb.cgi?T=JS&NEWS=N&PAGE=main&SHAREDSEARCHID=3ijyCeiCoEA4r5aC5uHUl6Xmj7ja9VLPz6tWDfHkZbi0S6ifx8kvKa7laRqdkQdCh>

1 breast cancer-related lymphedema/ 987

2 exp neoplasm/ and lymphedema/ 10324

3 ((lymphedema or lymphoedema or lymphodema or lymphedoema) and (cancer* or neoplas* or tumour* or tumor* or malignan* or melanoma* or sarcoma* or carcinoma* or metastas* or lymphoma* or axillary dissection* or lymph-node excision*)).mp. 13028

4 1 or 2 or 3 13777

5 exp compression therapy/ or exp compression garment/ or exp compression stocking/ or exp compression sleeve/ or exp compression bandage/ 18914

6 laser therapy/ or low level laser therapy/ 31035

7 shock wave therapy/ 2835

8 intermittent pneumatic compression device/ 1441

9 (compression or bandag* or wrapping or wraps or wrapped or pressure garments or stocking or stockings or lymph* sleeve* or pump or pumps or hose or hosier* or kinesiotap* or laser therap* or light therap* or ((extracorporeal or shock or shockwave or hifu) adj5 (therap* or treatment*))).mp. 563020

10 5 or 6 or 7 or 8 or 9 563178

11 4 and 10 1721

12 limit 11 to (english language and yr="2018 -Current") 612

13 limit 12 to (conference abstracts or "preprints (unpublished, non-peer reviewed)") 108

14 12 not 13 504

15 exp clinical trial/ or (randomi* or randomly or (random adj4 (allocat* or distribut* or assign*)) or placebo or trial or groups or subgroups or (phase adj1 ("3" or "2" or "1" or III or II or I))).tw. or rct.ti. 6124925

16 (pubmed or medline or cochrane or scopus or cinahl).tw. or (systematic* adj3 review*).mp. or meta-analy*.pt,mp. or (meta-analy* or meta analy* or metaanalys* or umbrella or research synthesis or overview).tw. or (technology assessment* or hta).mp. or review.pt. 3784677

17 ((case-control* or (cross-sectional not cross-sectional-area) or cohort or qualitative or (observational adj2 study) or case-series or case-report or case-study or delphi-study or bibliometric-analys* or questionnaire or survey or (tool and validat*)) not (trial or rct or review or metaanal* or meta-anal*)).ti. 1140298

18 (14 and (15 or 16)) not 17 243

**CINAHL Plus with Full Text (Ebscohost interface)**

Date searched: July 11, 2023 Results: 51

S1 ( (MH "Neoplasms+") AND (MH "Lymphedema") ) OR ( (lymphedema or lymphoedema or lymphodema or lymphedoema) and (cancer* or neoplas* or tumour* or tumor* or malignan* or melanoma* or sarcoma* or carcinoma* or metastas* or lymphoma* or axillary-dissection* or lymph-node-excision*) )

S2 (MH "Elastic Bandages") OR (MH "Compression Garments") OR (MH "Compression Therapy") OR (MH "Bandaging Techniques") OR (MH "Taping and Strapping") OR (MH "Kinesiotaping") OR compression or bandag* or wrapping or wraps or wrapped or pressure garments or stocking or stockings or lymph* sleeve* or pump or pumps or hose or hosier* or kinesiotap* or laser therap* or light therap* or ((extracorporeal or shock or shockwave or hifu) N5 (therap* or treatment*))

S3 ( ((MH "Clinical Trials+") OR (MH "Community Trials") or randomi* or "randomly" or ("random" N4 (allocat* or distribut* or assign*)) or "placebo" or "trial" or "groups" or "subgroups" OR or (phase N1 ("3" or "2" or "1" or III or II or I)) OR TI(RCT)) ) OR ( AB(pubmed or medline or cochrane or scopus or web-of-science or cinahl or search*) OR ((systematic* or evidence-based or scoping or umbrella or rapid or integrative) N3 (review* or overview*)) or technology-assessment or hta or meta-analy* or meta analy* or metaanalys* or meta-synthes* or research-synthesis OR PT(systematic review or meta analysis or meta synthesis) )

S4 TI((case-control* or (cross-sectional not cross-sectional-area) or cohort or qualitative or (observational N2 study) or case-series or case-report or case-study or delphi-study or bibliometric-analys* or questionnaire or survey or (tool and validat*)) not (trial or rct or review or metaanal* or meta-anal*))

S5 S1 AND S2 AND S3 NOT S4

**Limiters** - English Language; Published Date: 20180101-20231231

Supplementary Material: Figure 1a-e


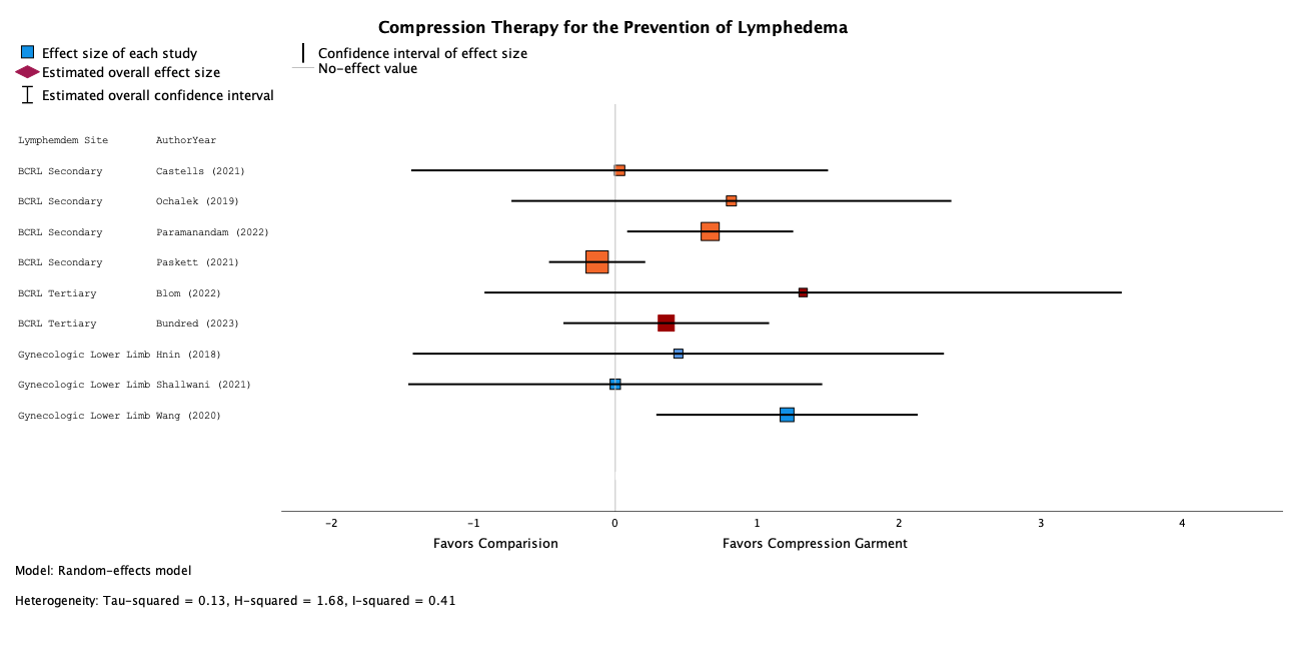

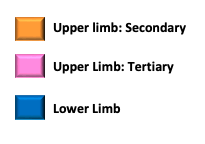


**Figure 1a: Trials in the Prevention Phase**


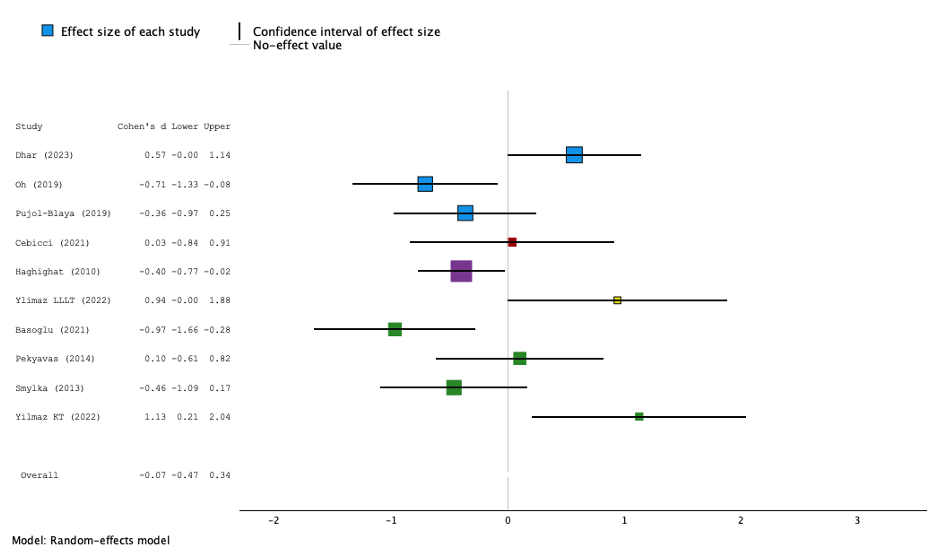

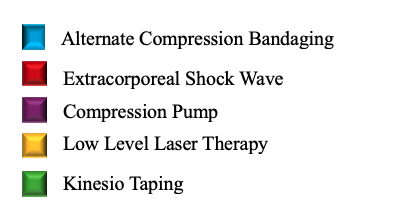


**Figure 1b: Trials examining Alternative Interventions in the Reduction Phase (N = 10 comparisons)**


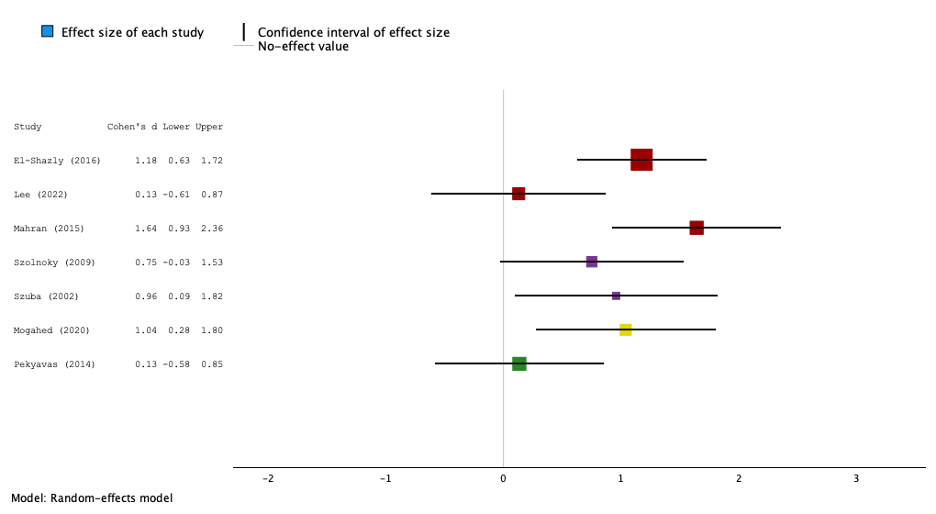

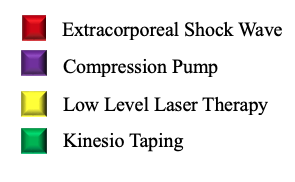


**Figure 1c: Trials examining Adjunctive Interventions in the Reduction Phase**

**
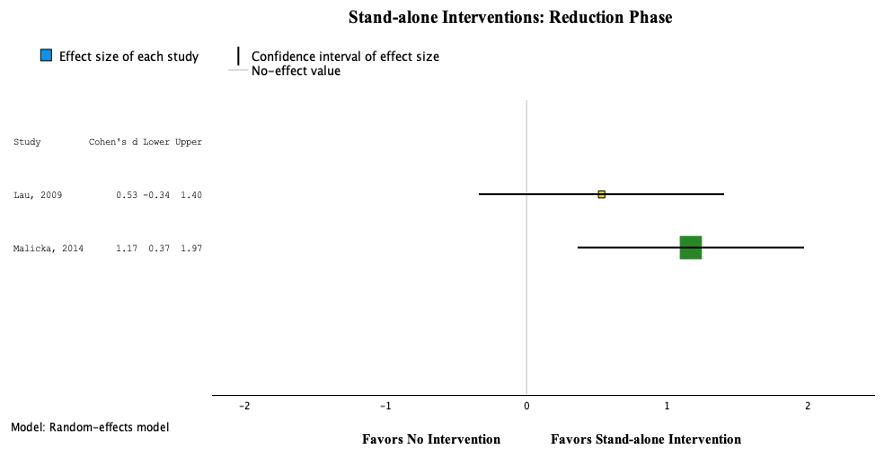
**
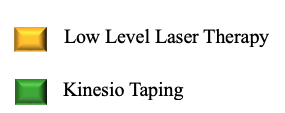


**Figure 1d: Trials examining Stand-alone Interventions in the Reduction Phase**

**
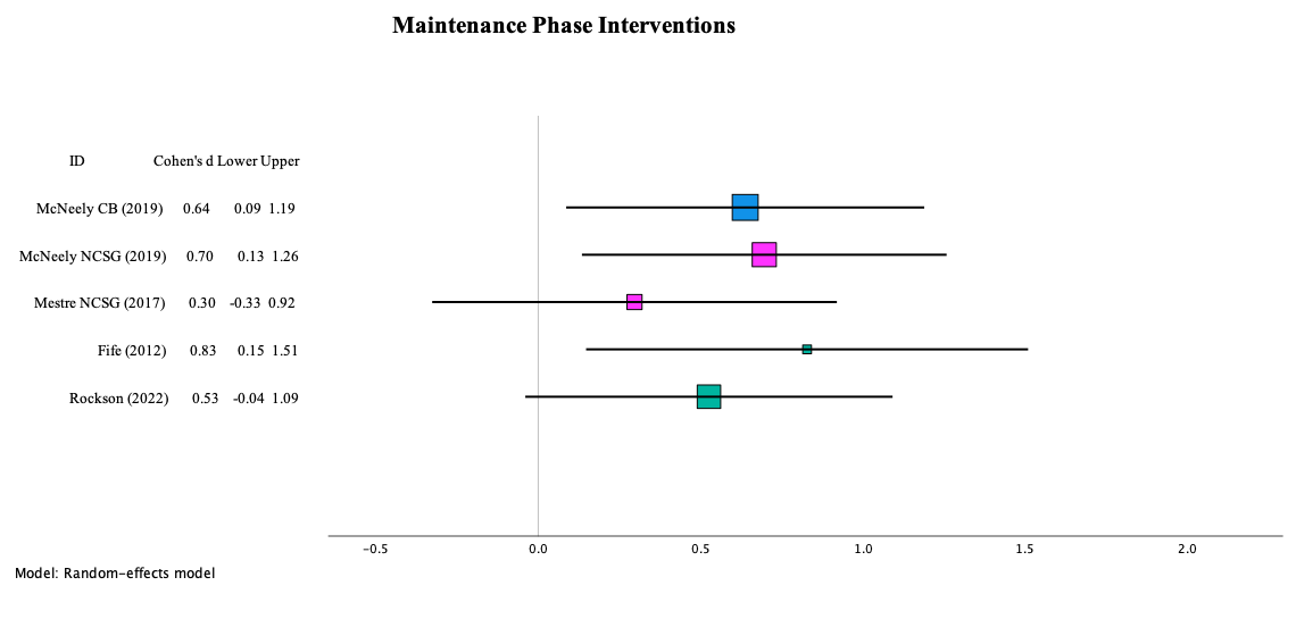
**
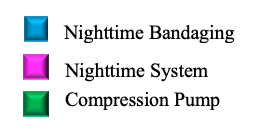


**Figure 1e: Trials examining Interventions in the Maintenance Phase**
